# Strategies against Burnout and Anxiety in Medical Education – Implementation and Evaluation of a New Course on Relaxation Techniques (*Relacs*) for Medical Students

**DOI:** 10.1371/journal.pone.0114967

**Published:** 2014-12-17

**Authors:** Katharina Wild, Michael Scholz, Axel Ropohl, Lars Bräuer, Friedrich Paulsen, Pascal H. M. Burger

**Affiliations:** 1 Department of Anatomy II, Friedrich-Alexander-University Erlangen-Nürnberg, Erlangen, Germany; 2 Hospital Meissenberg, Psychiatric and Psychotherapeutical Specialist Hospital for Women, Zug, Switzerland; University of Geneva, Switzerland

## Abstract

Burnout and stress-related mental disorders (depression, anxiety) occur in medical students and physicians with a significantly higher prevalence than in the general population. At the same time, the learning of coping mechanisms against stress is still not an integral part of medical education. In this pilot study we developed an elective course for learning relaxation techniques and examined the condition of the students before and after the course. 42 students participated in the semester courses in 2012 and 2013 as well as in a survey at the start and end of each course. The students were instructed in autogenic training (AT) and progressive muscle relaxation according to Jacobsen (PMR) with the goal of independent and regular exercising. At the beginning and the end of the semester/course the students were interviewed using standardized, validated questionnaires on burnout (BOSS-II) and anxiety (STAI-G), depression (BDI), quality of life (SF-12) and sense of coherence (SOC-L9). We compared the results of our students participating in *Relacs* with results from eight semester medical students (n = 88), assessed with the same questionnaires at similar points of time within their semester. Participating students showed a significant decline in cognitive and emotional burnout stress and in trait anxiety. Furthermore, they showed a reduction in state anxiety and a conspicuous decrease in mean depression. The sense of coherence increased at the same time. A comparative cohort of medical students of 8^th^ semester students, showed lower values for the specified measurement parameters at the beginning, but showed no progressive changes. Our course introducing AT and PMR led to a significant reduction of burnout and anxiety within the participating group of medical students. Even the course attendance for just one semester resulted in significant improvements in the evaluated parameters in contrast to those students who did not attend the course.

## Introduction

Compared with the average population, clinical physicians and medical students already suffer with significantly greater frequency from stress-associated mental disorders such as depression and anxiety disorders [1]. In a current survey of medical students in the preclinical semesters (n = 530), we found 40.8% students with an increase in anxiety and rates of elevated depressive symptoms as early as the fourth semester cohort. In 16.9% of the participating students this symptom showed clinically relevant severity [2]. The comparison of medical students at the beginning of the first semester and before the first state examination after only two years of study (4th semester) also showed a dramatic increase in cognitive and emotional burnout symptoms and a loss of mental quality of life as well as sense of coherence [2], [3]. This cannot be regarded as a local phenomenon, since similar changes have been observed in other national and international surveys as well [4]–[6]. In a recent review Ishak et al. stated that at least half of the medical students experience burnout in their time at university [7]. A progressive deterioration of these mental symptoms and disorders can also been found in study data from Switzerland, where among surveyed medical students of higher semesters and young doctors in training about 30% showed manifest depression and about 15% anxiety disorders [8]–[10].

In our opinion, one reason for the high number of existing mental illnesses and stress syndromes is insufficient formulation and teaching, or rather learning, of strategies for dealing with the day-to-day stress inherent in the medical profession. Medical students with active coping strategies basically deal with these difficulties more successfully [11]. On the other hand, work habits change over the course of a medical education and burnout risk behavior is more frequent with longer duration of medical education [12].

The incidence of stress-induced burnout increases the longer the student is in the medical education process and continues to increase in the medical assistant phase. Distress due to mental impairment increases the risk of medically incorrect decisions. Warnecke et al. postulate in their article of 2011 that it is necessary for the safety of physicians and patients for medical students to develop intervention strategies to counter these negative factors early on. "In a university medical degree course in which students are taught about managing the health of others, there is an imperative to provide them with effective, evidence-based ways to manage their own stress.“ [13].

For all the reasons mentioned, anchoring of stress management as an integral part of medical training strategies is therefore all the more necessary [14]. The efficacy of relaxation techniques to counter stress, anxiety and depression in general, and among medical students in particular, has been clearly established: techniques used in the care of psychiatric patients, such as mindfulness exercises, have already been successfully used on medical students and led to a reduction of anxiety and stress perception among the participants. This effect remained persistent even after the intervention was finished [13]. Even self-hypnosis, as a further relaxation technique, reduced the sense of distress among medical students and promoted positive effects [15]. Observation of students in different subjects revealed a positive impact of progressive muscle relaxation on anxiety and quality of life of those practicing the technique [16]. Relaxation and stress management techniques thus provide satisfactory alleviation of the massive mental stress encountered during medical education and decrease the risks of burnout, depersonalization, anxiety and stress among medical students [12], [17].

Against this background, we developed and offered our medical students a new elective course in autogenic training (AT) and progressive muscle relaxation (PMR) starting with the summer semester of 2012. We intended to test our hypothesis whether it is possible to integrate a successful, well accepted and effective course offer for medical students in order to improve their mental health. The course name *Relacs* is an acronym for "recreation and success in learning through applied concentrative self-relaxation". The data obtained on the participating students for burnout and anxiety were evaluated and analyzed and are presented in this paper.

## Material and Methods

A total of 39 medical students (classical curriculum, clinical section, 5th to 8th semester) and three psychology students (6th semester) participated in *Relacs* during the 2012 summer term and 2012/2013 winter term. The course took place in small groups (max. 12 students) during one semester and with one session (2h) per week and group. AT and PMR were taught in theory and practice. In addition to these sessions, independent practice units are required twice daily covering the contents of the course as taught and elaborated with relaxation exercises in AT or PMR. Psychometric data were collected from 11 students at one point in time and from 31 students at two points. Two participants in the first survey (summer term 2012) did not fully complete their questionnaires, so that not all parameters were analyzed for them. A total of 37 female and 5 male students aged 22–39 years (mean  = 24.93±4.42 years) participated in Relacs. For the second point of time in the survey, winter term 2012/13, 27 female and 4 male participants aged 20–33 years were interviewed (mean 24.44±3.14 years). Methods included proper consent and approval, complied with the declaration of Helsinki, and were approved by the ethic committee of the Friedrich-Alexander University (FAU), Erlangen, Germany. Further consent of the student participants was not given or necessary, because all data of this study were analyzed anonymously. All participants gave written consent to data collection and analysis of their test results. The statistical analysis was performed using the statistical software IBM SPSS Statistics.

Students were interviewed using standardized and validated psychological questionnaires on burnout (burnout screening scales; BOSS-II), anxiety (State and Trait Anxiety Inventory; STAI-G) and other parameters (depression, sense of coherence, work-related behavior and experience patterns). The surveys were collected at the same time for all students before and after the course, i.e. before the exam dates for other subjects at the end of the semester.

The burnout symptom scales (BOSS-II) are a self-assessment method for detection of subjective mental and physical symptoms that typically occur in the context of a burnout syndrome. Two independently applicable questionnaires with 30 items each are available. BOSS-II consists of three scales (physical, cognitive and emotional symptoms), each with ten items, covering an assessment period of seven days. This questionnaire can be used for both dimensional diagnostics (quantification of symptoms) and categorical diagnostics (diagnosis of burnout syndrome) [18].

The STAI-G is based on the distinction between anxiety as a state and anxiety as a trait [19]. The two scales of the STAI, with 20 items each, are used to collect anxiety state and trait data, respectively . The trait model of anxiety in the design of the STAI has been expanded to include the aspect of anxiety as a temporary emotional state of varying intensity as to time and situation (state anxiety). By contrast, anxiety as a relatively constant personality trait (trait anxiety), refers to individual differences in the tendency to anxiety reactions.

At the beginning of the semester, the students were interviewed concerning their work-related behavior and experience pattern (short form AVEM-44) [20], resulting in a classification into four types for this student cohort. Two of these behavior and experience types reflect a protective pattern, the other two a risk pattern with an increased risk for development of burnout and stress-related mental illness. For the assessment of depression and sense of coherence, two other mental parameters of interest, the Beck's Depression Inventory II [21] and the short form of the Sense of Coherence Scale SOC-L9 [22] were used. The corresponding results are covered respectively.

To generate a control group for the students participating in the *Relacs* course, other 8^th^-semester medical students were surveyed. To establish comparability of data acquisition times with the *Relacs* group, these assessments were held at the beginning (first week) of the semester and at the end of the semester (one week before final exams). The assessments were conducted with the same psychological questionnaires as listed above. In this control group, 61 female and 22 male students aged 22–40 years (mean  = 24.29±2.79) participated in the interviews.

## Results

Regarding participant age in the control and Relacs groups, no significant difference was found using the student's t-test for independent samples.

The analysis of work-related behavior and experience patterns data revealed in the Relacs and control groups revealed comparable constellations. Of the *Relacs* students, 61.3% showed a protective behavior and experience pattern (patterns S or G). In the control group, 57.8% of the students showed one of the two protective patterns. Patterns A and B, which are associated with an increased risk for stress-related disorders and/or burnout, were determined in 38.7% of the Relacs students, but in only 31.8% of the control group. 9.5% of the control group participants did not complete their questionnaires and were therefore excluded from analysis. The two groups thus appeared readily comparable with respect to work-related behavior and experience.

We found improved development in all Relacs students across all scales. The average depression level decreased, situational anxiety and basic anxiety decreased, the sense of coherence increased and burnout symptoms decreased in all three subscales considered (physical, cognitive and emotional; Table 1).

**Table 1:** Comparison of values from the psychological questionnaires at the start and end of the semester.

		MV	SD	SEM
**State-Anxiety**	start	45.40	5.04	0.92
	end	44.30	6.01	1.10
**Trait-Anxiety**	start	45.10	5.71	1.04
	end	42.30	5.06	0.92
**BOSS-II physical**	start	1.27	1.85	0.33
	end	0.83	0.60	0.11
**BOSS-II cognitive**	start	1.25	0.87	0.16
	end	0.92	0.72	0.13
**BOSS-II emotional**	start	0.91	0.79	0.14
	end	0.68	0.64	0.11

MV  =  mean value; SD  =  standard deviation; SEM  =  standard error of mean

**Figure 1:**
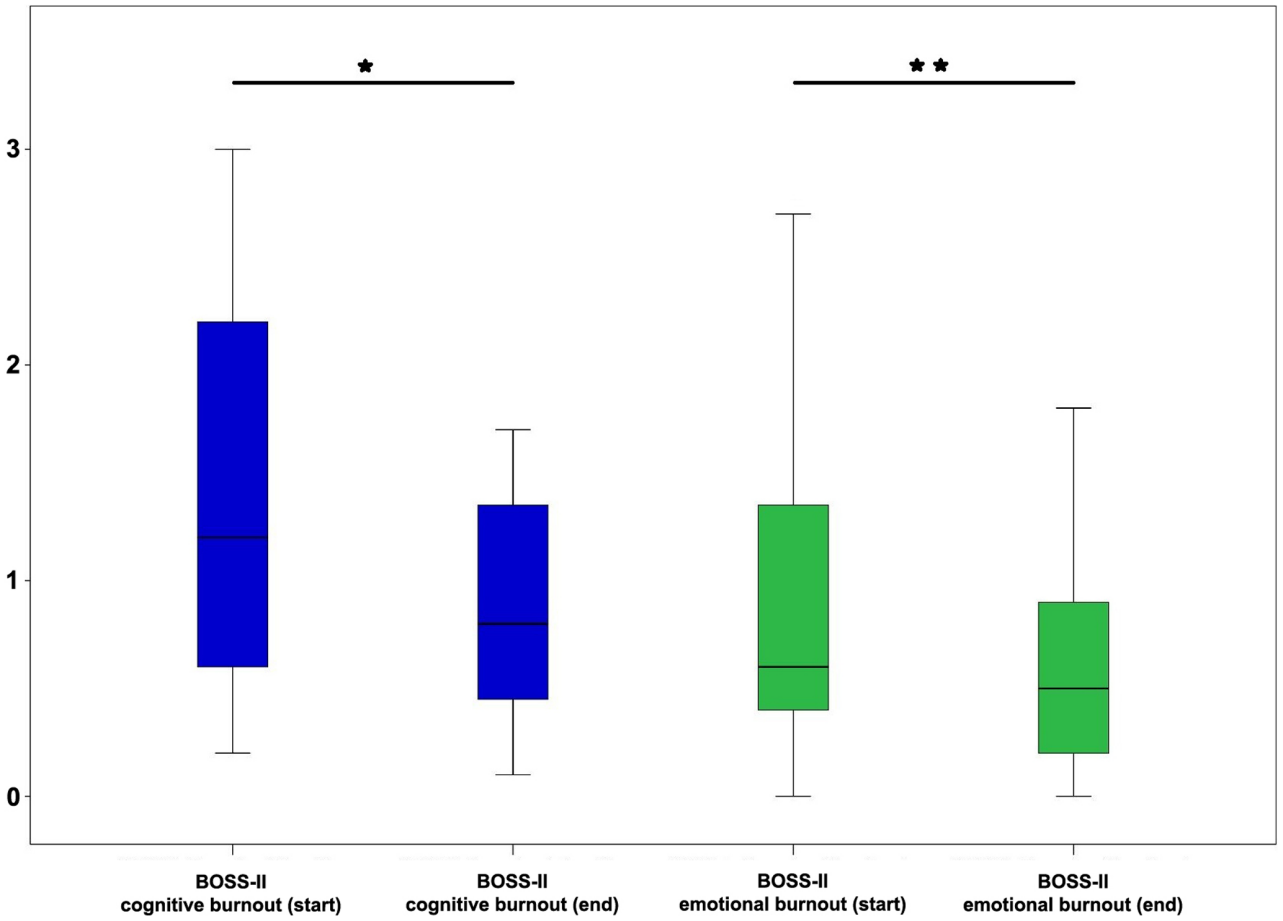
Comparison of values for physical, cognitive and emotional burnout symptoms in the course group at times of data collection start and end of semester. *  = p<0.05; **  = p<0.01

**Figure 2:**
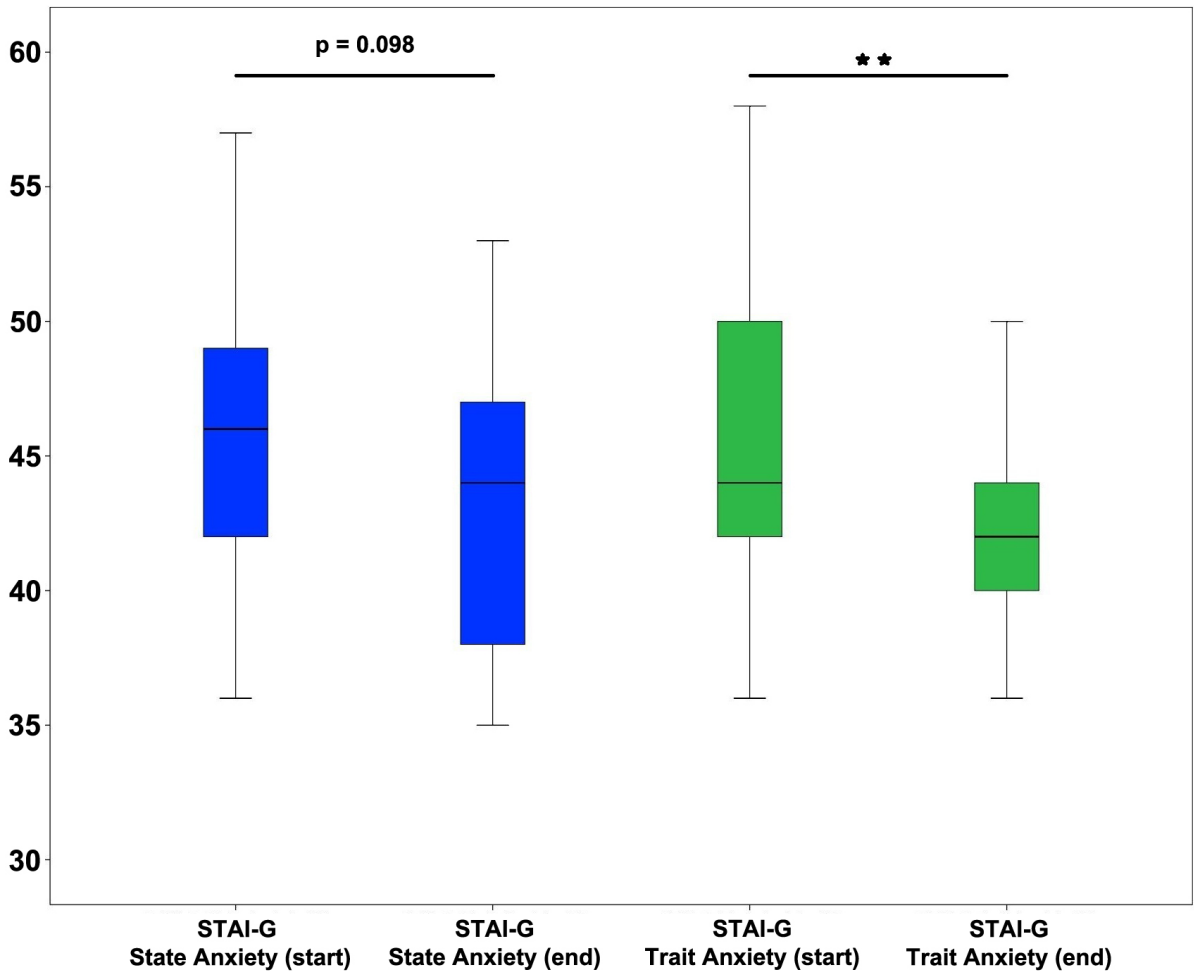
Comparison of values for state and trait anxiety in the course group at times of data collection start and end of semester. **  = p<0.01

Moreover, the reduction of trait anxiety as well as the decreases on the cognitive and emotional burnout scale were statistically significant (Table 2; Figs. 1, 2). Within the control group, on the other hand, no significant changes were determined in any of the parameters measured (Table 3).

**Table 2:** Composite t-test for the BOSS-II subscales and state and trait anxiety in the *Relacs* group, significant difference at p<0.05.

		Paired differences					t	p
		MV	SD	SEM	95% confidence interval for difference			
					Upper	Lower		
**State-Anxiety**	start-end	1.1	4.61	0.84	−0.62	2.82	1.31	0.20
**Trait-Anxiety**	start-end	2.80	5.14	0.94	0.88	4.72	2.99	0.01**
**BOSS-II physical**	start-end	0.45	1.86	0.33	−0.24	1.13	1.34	0.19
**BOSS-II cognitive**	start-end	0.34	0.81	0.15	0.04	0.63	2.31	0.03*
**BOSS-II emotional**	start-end	0.23	0.53	0.10	0.03	0.42	2.40	0.02*

MV  =  mean value; SD  =  standard deviation; SEM  =  standard error of mean; *  = p<0.05; **  = p<0.01

**Table 3:** Composite t-test for the BOSS-II subscales and state and trait anxiety in the control group, significant difference at p<0.05.

		Paired differences					t	p
		MV	SD	SEM	95% confidence interval for difference			
					Upper	Lower		
**State-Anxiety**	start-end	0.50	6.06	1.15	−1.85	2.85	0.44	0.67
**Trait-Anxiety**	start-end	0.93	4.48	0.86	−0.85	2.70	1.07	0.29
**BOSS-II physical**	start-end	0.09	0.43	0.08	−0.08	0.25	1.07	0.30
**BOSS-II cognitive**	start-end	0.35	1.75	0.33	−0.33	1.03	1.06	0.30
**BOSS-II emotional**	start-end	−0.08	0.54	0.10	−0.28	0.13	−0.74	0.47

MV  =  mean value; SD  =  standard deviation; SEM  =  standard error of mean

## Discussion

The results of our Relacs study as presented show for many parameters a significant effect of relaxation techniques in terms of reduction of burnout-associated discomfort and anxiety in participating students, most of whom were in the clinical segment of their medical education. Similarly collected data for a control group of medical students from the same period and at comparable time points showed no significant changes in the surveyed parameters.

It is noteworthy that, within the Relacs group, both trait and state anxiety had decreased by the second assessment. Although the reduction in state anxiety, i.e. situation-related anxiety, did not reach statistical significance (p = 0.20). This development should be seen within the time context of the semester – despite the upcoming exams at the end of the semester, both the students' basic anxiety levels and situational anxiety levels were reduced. This was not true of the medical students in the control group (8^th^ semester), who did not learn or apply relaxation techniques.

It proved possible to counteract clinical and subclinical burnout complaints from the emotional and cognitive range at the same time effectively. A slight, although not statistically significant, reduction was determined for physical complaints.

In general, the mental state of the *Relacs* students improved significantly during the course of the study. The results obtained in this pilot project are a clear indication for high efficacy of the learned relaxation techniques, especially in terms of effective burnout prevention, which can even be quickly learned parallel to regular clinical work. Such techniques are already established, and have proven effective, in many collectives [23]

We are aware of the limitations of the study population selection method used in this study due to the elective nature of the *Relacs* course without randomized participant selection. However, the study was not designed to provide general proof of efficacy of AT and PMR.

In their almost 100-year history, both techniques have been successfully used in various therapeutic regimens to treat somatic disorders, depression, anxiety and pain [24]–[26]. Based on the burnout prevention and anxiety reducing effects demonstrated in our study we contend that the students interested in learning stress management techniques (in our case AT and PMR) benefitted significantly from the *Relacs* course content.

Mandatory inclusion of relaxation techniques in an existing curriculum of basic medical studies cannot be advocated based on the study results alone. This would require a larger number of participants and a higher-grade study design with matched case control groups.

The fact, however, that in a given number of persons from a high-risk collective for development of stress-related mental disorders - and medical students can be considered to be in this category based on international survey results [27], [28] - such a clear effect was observable after only a one-semester accompanying course is remarkable and should be taken into account when establishing medical curricula. Within our cohort of 42 students positive effects were observed and the psychological parameters analyzed revealed coherent results, clearly demonstrating the high potential of the applied relaxation techniques.

Integration of protective technique training in medical studies has been advocated by other authors within the context of the increased mental workload in medical education [13], [17]. Ishak [7], [29] stresses the importance of burnout as a crucial factor in medical students due to its high prevalence. The continuation of burnout symptoms beyond medical school is real and the consequences for medical professionals and subsequently their patients can be severe, resulting in psychiatric disorders like depressions and problematic patient care [30], [31].

Of course there are several factors to be accounted for in order to work against the development of burnout in medical professionals, like personal support, work satisfaction [32].

There is still not enough awareness on the educators' side for a necessity to integrate the care for the medical student's own personal, mental health within the curriculum at medical schools [7]and thus prevent burnout and subsequent manifest psychiatric disorders.

The data presented shows one opportunity to approach the goal of preventing mental disorders and their development among medical students. Provision of opportunities - at least firstly for motivated students - to learn protective relaxation techniques integrated in the medical curriculum should be a future prerequisite for medical faculties. The participating Relacs students gained a certain level of awareness and reflection regarding the stressful and challenging character of their studies and the task of a professional physician that awaits them. At the same time they were (and are) motivated to address these issues more actively and achieved very good results by participating in *Relacs*.

In addition to the state of improvements as reported, the *Relacs* students gave this elective excellent grades (overall course evaluation according to the German school grading system (5 fail to 1 best) was 1.229 =  very good). In the evaluation process, the participants were very open-minded and motivated to learn protective relaxation techniques for their professional life, regardless of levels of interest and pre-educational training with respect to the course content (Table 4). Without exception, the students reported a strong belief in having learned something important and helpful.

**Table 4:** Extract from course evaluation of *Relacs* in summer semester 2012; 9 listed items corresponding to the six-level evaluation scale from 1 ( =  not at all) to 6 ( =  definitely true).

	Minimum	Maximum	MV	SD
**The important/benefit of the themes presented is communicated well**	5	6	**5.77**	0.43
**The theme of the course is relevant ** ***per se*** ** (professional/practical/examination)**	3	6	**4.73**	0.92
**I am learning a great deal in this course**	3	6	**4.77**	0.75
**My level of knowledge is much higher due to the course**	3	6	**4.62**	0.80
**My grasp of the subject is more fundamental now than before the course**	3	6	**5.27**	0.83
**I am learning something meaningful and important**	4	6	**5.19**	0.69
**The course enhances my interest in my studies**	2	6	**4.56**	1.00
**The course motivates me to take an interest in the content offered**	4	6	**5.00**	0.75
**The course is worth attending**	5	6	**5.69**	0.47
**Overall school grade for the course**	1.0	2.0	**1.23**	0.42

Twice previously, prior to the *Relacs* course reported on here, training in relaxation techniques (AT, PMR) were offered as a voluntary elective course to medical students in the higher clinical semesters. These courses were also given consistently positive evaluations by the students, whereby a subjective sense of success for the individual participant was always emphasized. This empirical impression has now been underlined and confirmed by the *Relacs* evaluation and data analysis.

Since AT and PMR are regularly used, established relaxation techniques in psychiatry at many locations worldwide, inclusion of adequate courses in medical curricular on a broad basis should be an easy matter. In addition to the positive effects already mentioned (burnout prevention, etc.), students were also taught didactically significant aspects of relevant treatment methods in the early stages of their medical training. The *Relacs* course was based on maximized use of communicative teaching opportunities, a particularly motivating aspect for the participants. The focus on the pursuit of learning and continuous exercising during the lessons also reflects the demand for more practice-oriented teaching in medical curricula.

Students are particularly receptive to the interdisciplinary practical knowledge imparted by the course due to the emphasis on the self-awareness aspect. They see the experience as stimulating and valuable for their medical studies as a whole (see details in Table 4).

In sum, the transfer of our concept to other universities would appears to be a simple matter. *Relacs* is cost effective and could be readily integrated into training settings to assist students to cope with stress. This is important as the structures of curricula differ extensively between universities and a course offer like ours is widely adaptable and should be integrated easily without financial or organizational problems.

Follow-up surveys of the participating Relacs students – in particular regarding sustainability of the positive effect – are pending and will be conducted in the coming semesters within the context of an advanced course as requested by the students.
